# Retinol binding protein 4 promotes hyperinsulinism-induced proliferation of rat aortic smooth muscle cells

**DOI:** 10.3892/mmr.2014.2028

**Published:** 2014-03-07

**Authors:** FEI LI, KE XIA, MD SAYED ALI SHEIKH, JINFANG CHENG, CHUANCHANG LI, TIANLUN YANG

**Affiliations:** 1 Department of Cardiology, Xiangya Hospital, Central South University, Changsha, Hunan 410008, P.R. China; 2 Center for Vascular Biology and Inflammation, Cardiovascular Division, Department of Medicine, Brigham and Women’s Hospital, Harvard Medical School, Boston, MA 02115, USA; 3 Department of Geriatrics, Xiangya Hospital, Central South University, Changsha, Hunan 410008, P.R. China

**Keywords:** retinol binding protein 4, hyperinsulinism, vascular smooth muscle cells, proliferation, signaling pathway

## Abstract

Recent studies have suggested that retinol binding protein 4 (RBP4), an adipocytokine related to insulin resistance (IR), may play an important role in the development of atherosclerosis and cardiovascular diseases (CVD). Abnormal proliferation and migration of vascular smooth muscle cells (VSMCs) is one of the most common causes of atherosclerosis. Hyperinsulinism promotes proliferation of VSMCs through the MAPK pathway. However, whether RBP4 is involved in insulin-induced proliferation of VSMCs leading to atherosclerosis remains unclear. In the present study, we evaluated the role of RBP4 and the potential relevance of signaling pathways in this process. Different concentrations of RBP4 (1 and 4 μg/ml) were added to rat aortic smooth muscle cells (RASMCs) during insulin-induced proliferation. The levels of cell growth signaling pathway proteins ERK1/2, p-ERK1/2, JAK2, p-JAK2, STAT3 and p-STAT3 were assessed by western blotting in order to identify the pathway(s) that are activated during insulin-induced proliferation. The specific inhibitors of ERK1/2 (PD98059) and JAK2 (AG490) were used to confirm our findings. Insulin induced proliferation of RASMCs in a concentration- and time-dependent manner, and increased the expression of ERK1/2, p-ERK1/2, JAK2, p-JAK2, STAT3 and p-STAT3 in a time-dependent manner. RBP4 enhanced insulin-induced proliferation of RASMCs and expression of p-ERK1/2 and p-JAK2. RBP4-induced proliferation of RASMCs was reduced by the ERK1/2 inhibitor, while it was unaffected by the JAK2 inhibitor. These results suggest that RBP4 mediates VSMC proliferation induced by insulin via activation of the MAPK pathway, and highlight RBP4 as a modulator of atherosclerosis in hyperinsulinemia, therby enhancing our understanding on a number of unexpected aspects of CVD.

## Introduction

The endocrine function of adipose tissue has been elucidated in recent years, with the identification of adipocytokines such as leptin, adiponectin and resistin. Adipose tissue produces numerous cytokines and hormones, which act as autocrine, paracrine and endocrine factors and participate in the pathophysiological process of insulin resistance (IR) and chronic inflammation (1,2). Retinol binding protein 4 (RBP4) is an adipocytokine related to IR (3). This was first shown in mice by Yang *et al* (3), who observed that knockout mice for the gene encoding adipose-specific glucose transporter-4 (GLUT-4) were insulin resistant in muscle and liver, and displayed increased expression of the *RBP4* gene. Graham *et al* (4) subsequently measured the serum RBP4 level, insulin resistance, and components of the metabolic syndrome in lean and obese individuals with or without type 2 diabetes, and found that the serum level of RBP4 correlates with insulin resistance. Additional studies further proved that the level of RBP4 in the blood associates with IR (5–8). IR is followed by compensatory hyperinsulinemia. It is widely accepted that hyperinsulinemia and insulin resistance are the main risk factors of cardiovascular diseases (CVD), eventually leading to the formation and development of atherosclerosis (9).

Vascular smooth muscle cells (VSMCs) have been extensively used to study the pathological mechanisms underlying atherosclerosis. Proliferation and migration of VSMCs is of important value for the formation of coronary atherosclerosis and the development of coronary heart disease (CHD). Insulin is a highly potent cell growth factor, which can promote VSMC proliferation and DNA synthesis, and plays an important role in the formation of atherosclerosis (10,11). Recently, RBP4, an adipocytokine related to IR, has been suggested to play an important role in the occurrence and development of atherosclerosis and CVD (12–14). However, whether RBP4 is involved in insulin-induced proliferation of VSMCs leading to atherosclerosis remains unclear.

Proliferation and migration of VSMCs are related to a variety of signal transduction pathways, such as the mitogen-activated protein kinase (MAPK) and the JAK/STAT pathway. Insulin activates the MAPK pathway through the Grb2/SOS and RAS proteins to promote cell growth and proliferation, and collagen synthesis (15–17). IR is followed by compensatory hyperinsulinemia, which promotes insulin-induced proliferation of VSMCs via the SHC/Raf/MAPK pathway, and accelerates artery atherosclerosis (18). JAK/STAT is another important signal transduction pathway mediating cell proliferation. Binding of cytokines such as interferon, 5-hydroxytryptamine, platelet-derived growth factor and others to the specific receptor activates the protein tyrosine kinase (PTK) Janus kinase (JAK), thereby activating signal transducer and activator of transcription (STAT), and inducing cell proliferation. Previous studies (19–21) have shown that the JAK/STAT signaling pathway plays an important role in VSMC proliferation.

Insulin promotes proliferation of VSMCs to induce formation of atherosclerosis through the MAPK pathway. Ost *et al* (22) examined the mechanisms of action of RBP4 in primary human adipocytes. RBP4-treated adipocytes displayed the same molecular defects in insulin signaling, mediated by the insulin receptor substrate (IRS) protein 1 and the MAP kinase, as adipocytes from patients with type 2 diabetes. Takebayashi *et al* (23) further showed that RBP4 has a robust acute effect on the enhancement of NO production via stimulating part of the PI3K/Akt/eNOS pathway and inhibiting insulin-induced ET-1 secretion, probably via the MAPK pathway, eventually causing vasodilatation. However, whether RBP4 is involved in insulin-induced proliferation of VSMCs leading to atherosclerosis remains unclear. In the present study, we evaluated the role of RBP4 in this process and the underlying signaling pathways.

## Materials and methods

### Reagents

RBP4 protein was purchased from Sino Biological Inc. (Beijing, China) and was dissolved in a solution comprising sterile 50 mM Tris, 10 mM CaCl_2_ and 150 mM NaCl at pH 7.5, at a final concentration of 500 μg/ml. Mouse anti-extracellular signal-regulated kinase (ERK)1/2 and -phospho-ERK1/2 (p-ERK1/2) monoclonal antibodies, and rabbit polyclonal anti-JAK2, -p-JAK2, -signal transducer and activator of transcription (STAT) 3 and -p-STAT3 antibodies were purchased from Santa Cruz Biotechnology, Inc. (Santa Cruz, CA, USA). HRP-conjugated goat anti-rabbit and anti-rat IgGs were purchased from ZSGB-Bio (Beijing, China). The specific inhibitors of ERK1/2 PD098059, and of JAK2 AG490, were purchased from Sigma-Aldrich (St. Louis, MO, USA). Supersignal West Pico Chemiluminescent substrate was purchased from Thermo Scientific (Rockford, IL, USA).

### Cell culture

The rat aortic smooth muscle cell (RASMC) line A10 was obtained from the American Type Culture Collection (Manassas, VA, USA). The cells were cultured in Dulbecco’s modified Eagle’s medium (DMEM) containing 10% fetal bovine serum (FBS), penicillin (100 U/ml), streptomycin (100 μg/ml) and NaHCO_3_ (3.7 g/l), in a humidified atmosphere of 5% CO_2_ at 37°C. Cells from passages 7 and 15 were used for the experiments.

### Cell proliferation assays

Cell proliferation was analyzed by using the MTT and a cell cycle assay. For the MTT assay, RASMCs were seeded in 96-well plates at a density of 0.6–1.0×10^4^ cells/well in DMEM supplemented with 10% FBS. After 24 h, the medium was replaced with DMEM containing 1% FBS to render the cells quiescent for 24 h. Then, cells were incubated for different time periods in DMEM containing 1% FBS and different concentrations of insulin. DMEM containing 1% FBS and RBP4 (1 or 4 μg/ml), PD098059 (5×10^−5^ M), or AG490 (5×10^−5^ M) was added to the cells, followed by treatment with insulin (10^−5^ M). Finally, 20 μl of 5 mg/ml MTT solution was added to each well and incubated for 4 h. The supernatants were aspirated, and the formazan crystals in each well were dissolved in 150 μl dimethyl sulfoxide. Cell proliferation was assessed by measuring the absorbance at 490 nm using a microplate reader (DTX800; Beckman Coulter, Brea, CA, USA).

For cell cycle analysis by flow cytometry, RASMCs were seeded in 6-well culture plates (1×10^5^ cells/well). After 24 h, the medium was replaced with DMEM containing 1% FBS to render the cells quiescent for 24 h. Then, RASMCs were pre-treated with RBP4 (1 or 4 μg/ml) for 1 h, or PD098059 (5×10^−5^ M)/AG490 (5×10^−5^ M) for 10 min, followed by treatment with insulin (10^−5^ M) for 24 h. After 24 h, cells were trypsinized and washed with phosphate-buffered saline (PBS) twice before fixing in ice-cold 70% ethanol at 4°C overnight. After removing ethanol by centrifugation, cells were washed with PBS and treated, for 20 min in the dark, with 50 μg/ml propidium iodide solution (Sigma-Aldrich) combined with 100 μg/ml DNase-free RNase, 0.1% Triton X-100 and 0.1 mM EDTA in PBS. After washing with PBS, propidium iodide-stained cells were subjected to cell cycle analysis using a FACScan flow cytometer (Becton Dickinson, Mountain View, CA, USA) and the FlowJo 7.1.0 software (Tree Star Inc., Ashland, OR, USA). At least 10,000 cells were counted for each sample. Data are presented as the percentage of cells in a given subpopulation.

### Western blotting

RASMCs were lysed in RIPA lysis buffer containing 0.1 M phenylmethylsulfonyl fluoride. After centrifugation at 12,000 rpm for 20 min, the protein concentrations of the supernatants were determined with the bicinchoninic acid assay (Sigma-Aldrich). Equal amounts of protein were mixed with sodium dodecyl sulfate (SDS) buffer and incubated at 100°C for 5 min before loading. Then, equal amounts of total protein were loaded and separated on a 15% SDS polyacrylamide gel by electrophoresis at a constant 90-V voltage for 2.5 h. Proteins were transferred under a standard module onto a polyvinylidene difluoride membrane (EMD Millipore, Billerica, MA, USA) for 45 min. The membrane was blocked with a 5% dry milk/Tris-buffered saline-Tween-20 (TBST) solution for 1 h at room temperature, and then probed with primary antibodies (anti-ERK1/2, -p-ERK1/2, -JAK2, -p-JAK2, -STAT3, -p-STAT3, dilution 1:1,000; -β-actin, 1:3,000) in a 5% dry milk/TBST solution overnight at 4°C. The membrane was rinsed several times with TBST buffer and then incubated with HRP-conjugated secondary antibodies (1:5,000) for 1 h at room temperature. Excess secondary antibody was removed by 3–4 washes in TBST buffer and the targeted protein bands were detected using an enhanced chemiluminescence (ECL) detection kit, followed by exposure of the membrane to an X-ray film. Protein bands were scanned and quantified using Image J software version 1.40 (National Institutes of Health, Bethesda, MD, USA). The quantified data for each protein were normalized to those for β-actin.

### Statistical analysis

Data are expressed as means ± SD and were statistically analyzed by Student’s paired t-test for pairwise comparisons or ANOVA followed by Newman-Student-Keuls test for comparisons among multiple groups. P<0.05 was considered to indicate a statistically significant difference. The SPSS 13.0 software was used for the analyses (IBM, Armonk, NY, USA).

## Results

### Proliferation of RAVSMCs induced by insulin

**Figure 1:**
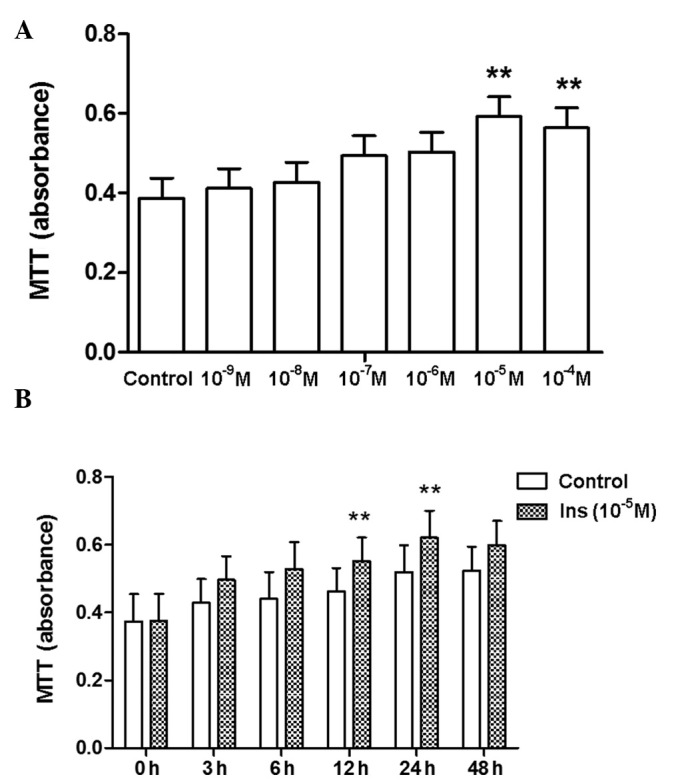
Proliferation of rat aortic smooth muscle cells (RASMCs) induced by insulin is (A) concentration-dependent and (B) time-dependent, with a peak observed at 24 h. Proliferation was determined using the MTT assay. Data come from 8–10 independent experiments. ^**^P<0.01 vs. control (0 h/0 M).

As previously mentioned, hyperinsulinemia is considered an independent risk factor for atherosclerosis and CVD. Insulin can promote VSMC proliferation and DNA synthesis, playing an important role in the formation of atherosclerosis. We therefore used insulin to stimulate proliferation *in vitro*. In line with results from a previous study (24), insulin induced proliferation of RASMCs in a concentration- and time-dependent manner, as shown by the increase in formazan absorbance (Fig. 1) and in the proportion of cells detected at the S and G2 phases (data not shown).

### Expression of ERK1/2, p-ERK1/2, JAK2, p-JAK2, STAT3, p-STAT3 in RASMCs is induced by insulin

**Figure 2:**
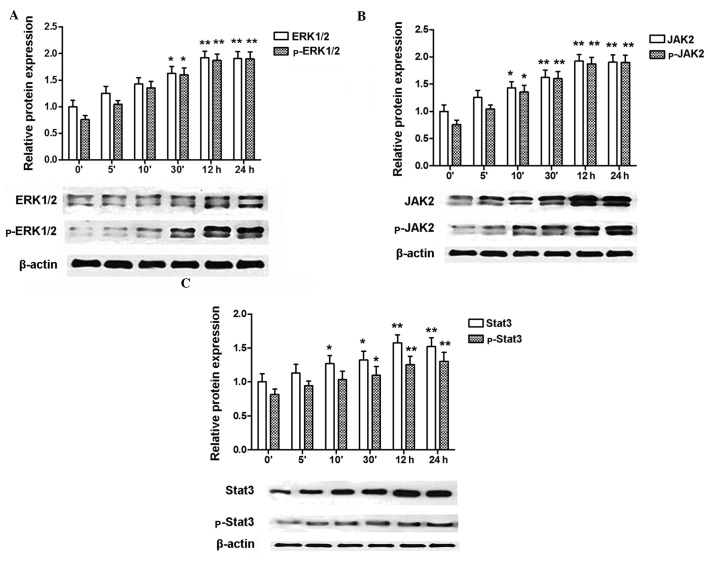
Expression of ERK2, p-ERK2, JAK2, p-JAK2, STAT3, p-STAT3 in rat aortic smooth muscle cells (RASMCs) induced by insulin. (A) Insulin induces the expression of ERK1/2, p-ERK1/2 in a time-dependent manner, with the maximum level of both proteins detected at 24 h. (B) Insulin induces the expression of JAK2, p-JAK2 in a time-dependent manner, with the maximum level of JAK2 detected at 12 h, and of p-JAK2 at 24 h. (C) Insulin induces the expression of STAT3, p-STAT3 in a time-dependent manner, with the maximum level of STAT3 detected at 12 h, and of p-STAT3 at 24 h. Blots are representative of three independent experiments. ^*^P<0.05, ^**^P<0.01 vs. control (0′ without insulin).

Since insulin induced proliferation of RASMCs in a concentration- and time-dependent manner, we used the most effective concentration (10^−5^ M) of insulin to further study the activation of signal transduction pathways related to cell proliferation. Insulin treatment enhanced the protein levels of ERK1/2, p-ERK1/2, JAK2, p-JAK2, STAT3, and p-STAT3 in a time-dependent manner, as shown by western blotting analysis (Fig. 2).

### RBP4 enhances insulin-induced proliferation of RASMCs

**Figure 3:**
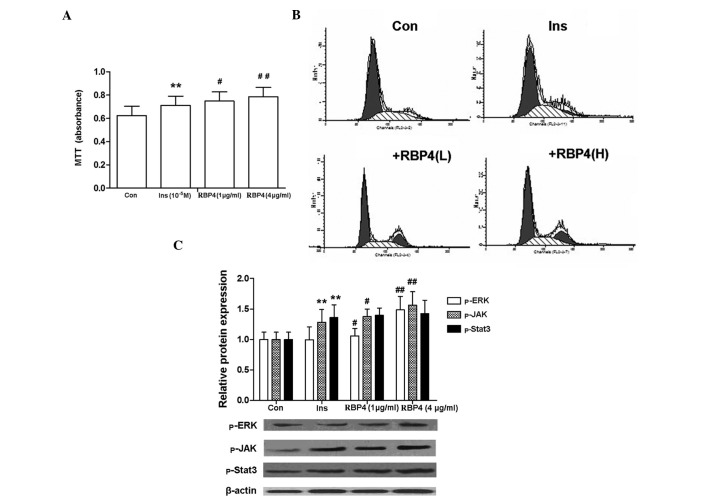
Retinol binding protein 4 (RBP4) increases insulin-induced proliferation of rat aortic smooth muscle cells (RASMCs), as determined by (A) the MTT assay and (B) flow cytometry. (C) RBP4 increases the protein level of p-ERK and p-JAK, but not that of p-STAT3. The blot is representative of three independent experiments. ^**^P<0.01 vs. Con; ^#^P<0.05, ^##^P<0.01 vs. Ins. Con, untreated cells; Ins, cells treated with 10^−5^ M insulin for 24 h; +RBP4(L), cells pre-treated with RBP4 (1 μg/ml) for 1 h prior to treatment with insulin; +RBP4(H), cells pre-treated with RBP4 (4 μg/ml) for 1 h prior to treatment with insulin. Data come from 8–10 independent experiments.

RBP4 enhanced the insulin-induced proliferation of RASMCs, as shown by the increase in formazan absorbance and in the proportion of cells at the S + G2 phases (Fig. 3A and B). The phosphorylation of ERK1/2, JAK2 and STAT3 is an indicator of the activation of the MAPK and the JAK/STAT signal transduction pathways. Notably, the expression of p-ERK and p-JAK2 that was induced by insulin was further enhanced by RBP4 treatment, while that of p-STAT3 remained unchanged (Fig. 3C).

### Involvement of ERK1/2 and JAK2/STAT3 pathways in the enhancement of RASMC proliferation by RBP4

**Figure 4:**
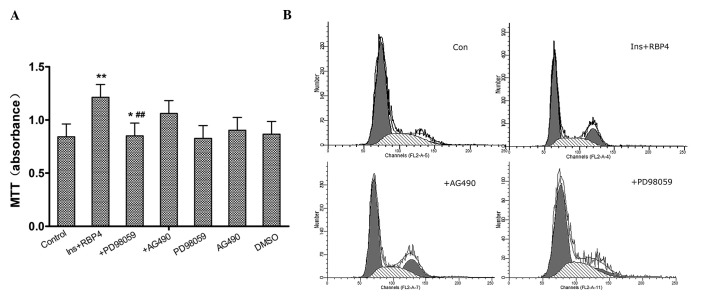
Involvement of the ERK1/2 and JAK2/STAT3 pathways in retinol binding protein 4 (RBP4)-mediated enhancement of rat aortic smooth muscle cell (RASMC) proliferation. (A) Proliferation of RASMCs measured by the MTT assay. RBP4-induced proliferation of RASMCs is inhibited in the presence of the JAK1/2 inhibitor PD98059 (5×10^−5^ M) but not of the JAK2 inhibitor AG490 (5×10^−5^ M). Data come from 3–8 independent experiments. ^*^P<0.05, ^**^P<0.01 vs. Con; ^##^P<0.01 vs. Ins (10^−5^ M) + RBP4 (4 μg/ml). (B) Cell cycle analysis by flow cytometry. Control, untreated cells; Ins + RBP4, cells treated with 10^−5^ M Ins + RBP4 (4 μg/ml) for 24 h; +PD98059, cells pre-treated with PD98059 (5×10^−5^ M) for 10 min prior to treatment with insulin and RBP4; +AG490, cells pre-treated with AG490 (5×10^−5^ M) for 10 min prior to treatment with insulin and RBP4; DMSO, wild-type cells treated with 0.1% DMSO (PD98059 isovolumetric).

To determine whether the ERK1/2 and/or the JAK2/STAT3 pathway are involved in the enhancement of insulin-induced cell proliferation caused by RBP4, PD98059 (the specific ERK1/2 inhibitor) and AG490 (the specific JAK2 inhibitor) were used. As shown by the MTT assay and flow cytometry, pre-treatment of cells with PD98059 (5×10^−5^ M) significantly (P<0.01) inhibited proliferation of RASMCs, while pre-treatment with AG490 (5×10^−5^ M) had no effect on proliferation (Fig. 4).

## Discussion

The present study examined the effect of RBP4 on insulin-induced proliferation of RASMCs. The main findings are the following: i) insulin induced proliferation of RASMCs via the MAPK and JAK2/STAT3 pathways; ii) RBP4 enhanced insulin-induced proliferation of RASMCs and iii) RBP4 enhanced insulin-induced proliferation of RASMCs via the MAPK, but not the JAK2/STAT3 pathway. Collectively, these findings indicate that RBP4 may play an important role in the mediation of signals related to proliferation of VSMCs induced by insulin, which suggests that RBP4 may contribute to vascular remodeling in hyperinsulinemia.

Since the first study of Yang *et al* (3) on RBP4 effects in mice, related studies on RBP4 and insulin resistance, obesity, type 2 diabetes, and CHD have provided contradictory results. Mahmoudi *et al* (11) did not find a significant difference in the RBP4 level between a non-diabetic population with CHD and healthy controls. Mallat *et al* (12) attempted to prove that RBP4 can be used as a predictor of CHD. The authors studied 1,036 patients with CHD in a period of six years, and found that the risk of CHD increased in cases of increased RBP4 level. However, following adjustment of the data for other CHD risk factors, the positive correlation between the RBP4 level and the risk of heart disease proved not to be statistically significant, thereby suggesting that RBP4 may not be a robust predictor of CHD. However, there are studies that showed that RBP4 positively and significantly correlates to carotid intima-media thickness, which is an atherosclerosis indicator, and negatively correlates to flow-mediated dilatation (25,26). Independent correlations between RBP4 and the low-density lipoprotein or the oxidized low-density lipoprotein have also been reported (27,28). In addition, Cabré *et al* (29) showed that the RBP4 level is significantly higher in atherosclerotic patients with primary type 2 diabetes mellitus than in those without atherosclerosis. Overall, a potential correlation between RBP4 and atherosclerosis and CHD can not be unequivocally claimed. Atherosclerosis is the basic pathogenetic cause of CHD, while abnormal proliferation and migration of VSMCs is one of the most common causes of atherosclerosis, vascular restenosis and other CVDs.

Binding of insulin to insulin receptors (InsR) in the target organs and tissues activates the MAPK pathway through the Grb2/SOS and RAS proteins, thereby regulating gene transcription to promote cell growth and proliferation. In IR, even when pancreatic β cells show a good function, compensatory hyperinsulinemia occurs and promotes insulin-induced proliferation of VSMCs via the SHC/Raf/MAPK pathway; in these conditions, hyperinsulinemia accelerates arterial atherosclerosis.

In agreement with a previous study (24), we showed that insulin induces proliferation of RASMCs in a concentration-dependent manner, with a 10^−5^ M concentration exerting the strongest effect. We further showed that insulin induces expression of ERK1/2 and p-ERK1/2 in a time-dependent manner, and that this effect is the strongest at 24 h of treatment. The present and previous studies overall confirm that treatment with high concentrations of insulin stimulates RASMC proliferation through activation of MAPKs. Notably, we also observed a time-dependent stimulation of the expression of JAK, p-JAK, STAT3 and p-STAT3 by high concentrations of insulin in RASMCs. This result strongly suggests that the JAK/STAT cell proliferation signal transduction pathway is also regulated by high concentrations of insulin. Therefore, JAK/STAT pathways not only mediate proliferation of VSMCs induced by IL-6 (19), platelet-derived growth factor BB (30) and IL-18 (31), but may also have a role in hyperinsulinism-induced atherosclerosis.

In the present study, RASMCs were pre-incubated with RBP4 (1 or 4 μg/ml) for 1 h prior to treatment with 10^−5^ M insulin, and these experiments demonstrated that RBP4 significantly increases the ability of insulin to induce smooth muscle cell proliferation. The percentage of RBP4-treated cells that were detected at the S and G2 phases was considerably higher than that of the control cells. This study also found that RBP4 significantly enhances the insulin-induced level of p-ERK and p-JAK proteins, from which we can infer that the activation (phosphorylation) of ERK and JAK is significantly enhanced. No significant effect on STAT3 phosphorylation was observed under these conditions, thus we conclude that RBP4 enhances insulin-induced smooth muscle cell proliferation via the MAPK pathway. Pre-treatment of RASMCs with the ERK1/2 inhibitor PD98059 effectively inhibited the observed effects of RBP4, including cell cycle changes. However, treatment with the JAK inhibitor AG490 had no significant effect on RBP4-induced cell proliferation, and cell cycle distribution did not change. These findings argue for the involvement of the MAPK pathway in the promotion of the insulin-induced proliferation of RASMCs by RBP4.

Binding of insulin to the insulin receptor (InsR) mediates, via a number of signal transduction pathways, a variety of physiological effects in the target organs and tissues. There are at least two insulin-related signal transduction pathways: one is the phosphatidylinositide 3 kinase (PI-3K) pathway, which is activated by IRS; the second is the MAPK pathway, which is activated via the Grb2/SOS and RAS proteins. A high level of plasma RBP4 can enhance insulin resistance through inhibition of IRS-1 and activation of phosphatidylinositol 3-kinase (PI3-K) in skeletal muscles (3). In agreement with a study (22) reporting that RBP4 reduces insulin-induced phosphorylation of IRS-1 and ERK1/2 in human adipose tissue, Takebayashi *et al* (23) found that RBP4 increases the production of NO by stimulating part of the PI3K/Akt/eNOS pathway in vascular endothelial cells, and leads to vasodilation through inhibition of phosphorylation of ERK1/2 and insulin-induced secretion of endothelin. However, in the present study, RBP4 was shown to enhance phosphorylation of ERK1/2 in RASMCs, thereby promoting the proliferation of smooth muscle cells, and this effect was attenuated by the specific inhibitor of ERK1/2 PD98059. In target tissues and organs of individuals with obesity and type 2 diabetes, the IRS/PI-3K pathway is impaired, but the Shc/Raf/MAPK pathway remains intact, and is even stimulated (18). This ‘selective insulin resistance’ phenomenon mitigates insulin metabolic regulation, including its antagonistic effect in atherosclerosis. RBP4 can inhibit phosphorylation of IRS-1 (3,22), then promote selective insulin resistance to enhance insulin-induced proliferation of vascular smooth muscle cells, and synthesis of collagen and growth factors.

In conclusion, the present study showed that insulin induces proliferation of RASMCs in a concentration- and time-dependent manner via the MAPK and JAK2/STAT3 pathways. In addition, we report, for the first time to the best of our knowledge, that RBP4 enhances RASMC proliferation induced by insulin via activation of the MAPK signaling pathway. Since this is a novel finding, additional research is needed to confirm it and further investigate the underlying mechanisms. The results from the present and previous studies indicate that RBP4 has a prominent role as a modulator of atherosclerosis in hyperinsulinemia, and may contribute to a better understanding of the numerous unexpected aspects of CVDs.
